# Influence of riverine input on the growth of *Glycymeris glycymeris* in the Bay of Brest, North-West France

**DOI:** 10.1371/journal.pone.0189782

**Published:** 2017-12-20

**Authors:** Amy M. Featherstone, Paul G. Butler, Melita Peharda, Laurent Chauvaud, Julien Thébault

**Affiliations:** 1 Universite de Bretagne Occidentale, Institut Universitaire Européen de la Mer, Laboratoire des sciences de l’environnement marin (LEMAR UMR 6539 CNRS/UBO/IRD/Ifremer), Plouzané, France; 2 Université de Bretagne Occidentale, Institut Universitaire Européen de la Mer, Laboratoire des sciences de l’environnement marin (LEMAR UMR 6539 CNRS/UBO/IRD/Ifremer), Plouzané, France; 3 College of Life and Environmental Sciences, University of Exeter, Penryn, Cornwall, United Kingdom; 4 Institute of Oceanography and Fisheries, Split, Croatia; Universidade de Aveiro, PORTUGAL

## Abstract

A crossdated, replicated, chronology of 114 years (1901–2014) was developed from internal growth increments in the shells of G*lycymeris glycymeris* samples collected monthly from the Bay of Brest, France. Bivalve sampling was undertaken between 2014 and 2015 using a dredge. In total 401 live specimens and 243 articulated paired valves from dead specimens were collected, of which 38 individuals were used to build the chronology. Chronology strength, assessed as the Expressed Population Signal, was above 0.7 throughout, falling below the generally accepted threshold of 0.85 before 1975 because of reduced sample depth. Significant positive correlations were identified between the shell growth and the annual averages of rainfall (1975–2008; r = 0.34) and inflow from the river Elorn (1989–2009; r = 0.60). A significant negative correlation was identified between shell growth and the annual average salinity (1998–2014; r = -0.62). Analysis of the monthly averages indicates that these correlations are associated with the winter months (November–February) preceding the *G*. *glycymeris* growth season suggesting that winter conditions predispose the benthic environment for later shell growth. Concentration of suspended particulate matter within the river in February is also positively correlated with shell growth, leading to the conclusion that food availability is also important to the growth of *G*. *glycymeris* in the Bay of Brest. With the addition of principle components analysis, we were able to determine that inflow from the River Elorn, nitrite levels and salinity were the fundamental drivers of *G*. *glycymeris* growth and that these environmental parameters were all linked.

## Introduction

Annually-resolved palaeoenvironmental archives such as tree-rings [1, 2, 3] ice cores [4, 5, 6] and corals [7, 8] have provided valuable insights into the terrestrial and tropical marine environments of the past. Up until the last decade, however, no proxy archive with a similarly high resolution had been developed for the marine environment of the mid and high latitudes [9]. During the past decade, with the use of the shells of long-lived bivalve molluscs, in particular *Arctica islandica* [10, 11, 12], *Panopea abrupta* [13, 14, 15], and *Glycymeris spp*. [16, 17, 18], this gap in knowledge has begun to close. These species, as well as others, can be used to build extended decadal to multicentennial annual-resolved chronologies using techniques established within the field of dendrochronology [12]. These chronologies, especially when they can be precisely dated by being anchored in time with shells of a known date of death, provide a stratigraphy for geochemical and increment width proxies which, when successfully calibrated against instrumental series, can be used for palaeoenvironmental reconstructions at an annual resolution [19].

Growth increments found in the shells can provide useful information about the biology, ontogeny and environment of the individual and its population [20, 21, 22]. These increments can be formed regularly, for example as annual or tidal increments, or intermittently as a result of disturbance events that cause metabolic stress to the animal [20]. Endogenous growth rhythms have been found in a number of marine bivalve species. For example, tidal growth increments in shell formation have been identified in *Chione fluctifraga* and *C*. *cortezi* and are characterized as corresponding to emersion–immersion cycles [23]. Other species exhibit reduced or even interrupted shell growth during gametogenesis due to the allocation of energetic resources towards the production of gametes, such as *Crassostrea gigas* [24]. In other examples, such as the Antarctic bivalve *Yoldia eightsi*, endogenous cyclicity in growth has been observed for which there is no clear explanation [25].

Growth increment widths have also been successfully used to reconstruct seawater temperatures [15, 26] and climate oscillations [27]. More intermittent links with seawater temperatures were described by Butler et al [10] and Marali and Schöne [28]. In addition, other studies have found relationships between bivalve growth and various other environmental variables. For example, Helama et al [29] and Mette et al [30] found that *A*. *islandica* shell growth was related to the winter NAO, Gutierrez-Mas [31] found *Glycymeris spp*. fossils to be responding to sea-level rise and Bušelić et al [32] correlated the growth of *G*. *bimaculata*, in part, with the salinity of the surrounding waters.

The focus of this study is on *Glycymeris glycymeris* which is another species that has been used for reconstruction of past climatic and environmental variables [16, 26, 33]. *G*. *glycymeris* is a fairly large bivalve with a maximum length of 80 mm [26] that inhabits the north eastern Atlantic continental shelf from Cape Verde to Norway in water depths of up to 100m in areas with strong bottom currents [34]. Previous research has shown (i) that the periodic growth increments in *G*. *glycymeris* are formed annually and are synchronous within populations [16, 26, 33] and (ii) that individuals can live for nearly 200 years [16, 35]. This species is therefore an ideal proxy archive for marine paleoenvironmental studies as it can provide replicated and crossdated chronologies of shell material, with annual resolution and absolute dating. If sufficient quantities of suitable fossil material are available it will be possible to extend these chronologies back through several centuries.

The study site is in the Bay of Brest, a semi-enclosed mixed marine/estuarine ecosystem with an area of 180 km^2^ and an average depth of 8 m. The Bay of Brest is connected to the Atlantic shelf (Iroise Sea) by a strait to the west that is 2 km wide and 40 m deep, and is fed by two rivers. The larger river, the Aulne, has a catchment area of 1822 km^2^ and the smaller Elorn has a catchment of 280 km^2^. These two rivers provide up to 85% of the total freshwater input into the bay [36]. It is therefore a suitable region for an investigation into the influence of river inflow upon the hydrography, biological and environmental dynamics within the bay.

A major difference between the open ocean and semi-enclosed bays has to do with the dynamics of seasonal phytoplankton blooms [37]. Increasing nutrient loading during the last decade, resulting from intensifying agricultural practice, has focused attention on the possible trend towards coastal eutrophication. Estuarine ecosystems, particularly those in enclosed or semi-enclosed bays, are subject to high nutrient loading, but each bay responds differently to such inputs [38]. While the emergence of eutrophic conditions is characteristic of some coastal areas, others, including the Bay of Brest, have not yet exhibited such a critical evolution [39].

A typical configuration of factors drive biogeochemical dynamics in such semi-enclosed bays: density stratification is potentially induced by aperiodic river discharges, but stirring by tidal currents and wind prevents sustained vertical stratification in these partially mixed waters [39]. At the other end of the system, hydrodynamic exchanges with the open ocean limits the accumulation of organic matter [40]; however, intermittent inputs by river flow can maintain nutrient availability even after periods of high consumption [41].

The objective of this study is to create an extended and annually resolved *G*. *glycymeris* chronology and to evaluate its potential as an environmental indicator and archive of proxy records for past climatic and hydrographic variability. In order to achieve this objective, the following goals were set; (i) to determine the feasibility of crossdating live- and dead-collected shell material using *G*. *glycymeris* samples from the Bay of Brest, north-west France, (ii) to establish that these samples can be used to build a well-replicated and statistically robust chronology, (iii) to identify relationships between environmental factors and the growth of *G*. *glycymeris*.

## Methods

### Sample collection

**Fig 1:**
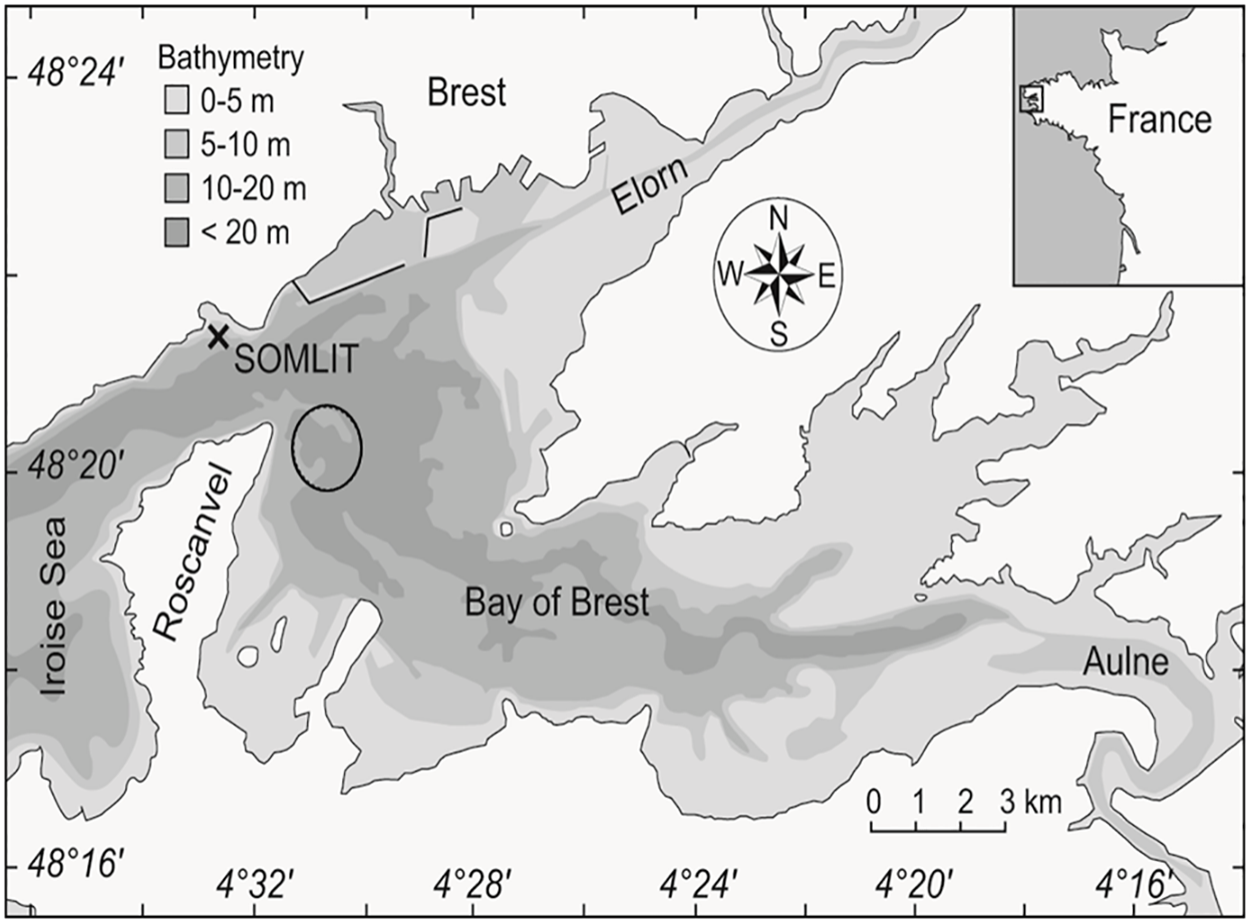
Location of Brittany in France and sample location within Brittany. Circle indicates sample site and ‘X’ shows the position of SOMLIT-Brest research station. Adapted from Thébault & Chavaud [42].

Sampling permit was given by the director of the Interregional Directorate for the Sea (North Atlantic—Western English Channel) on behalf of the Prefect of Brittany Region (sampling permit n°101/2014). Our study site, the Banc de la Cormorandière (48°20′26˝ N, 4°30′44˝ W), is a subtidal dune situated 20 m below the surface. It is located in the western part of the Bay of Brest, near its outlet and is subject to strong tidal currents (Fig 1). Living *G*. *glycymeris* and dead shells were collected monthly between September 2014 and November 2015. In total 401 live specimens and 243 articulated paired valves from dead *G*. *glycymeris* were collected in 20-25m water depth using a dredge deployed from the RV *Albert Lucas* (future access to these shells can be obtained through Dr. Julien Thébault (julien.thebault@univ-brest.fr)). Of the 243 pairs of valves from dead specimens, 114 were excluded from further consideration because of extensive bioerosion. A total of 14 shells live-collected from the same site in 2012 and archived at Université de Bretagne Occidentale were also used in the analysis.

Seawater temperature and salinity have been monitored weekly from 1998 to present at the SOMLIT-Brest station located at the outlet of the Bay of Brest (48°21′30” N, 4°33′06” W), using a Sea-Bird SBE 19 CTD profiler (Sea-Bird Electronics, Inc.). The sampling station, which is open to oceanic influence, is located in front of Ste-Anne-du-Portzic at the boundary between the Iroise Sea and the Rade de Brest (Fig 1). Sampling is carried out weekly, at 2 m depth, and five physico-chemical factors are measured: temperature, conductivity, dissolved oxygen, pH and turbidity, as well as a biological parameter (fluorescence of chlorophyll α).

In addition, the buoy MAREL-Iroise provides automatic records of temperature, conductivity, dissolved oxygen, pH, and turbidity measured every 20 min at a location 50m from the SOMLIT-Brest station (48°21’28”N, 4°33’05” E). These monitoring stations are located less than 3 km away from the shell sampling site.

### Shell preparation

The morphometrics (shell length, height, width, and total dry shell mass) of all the collected shells were measured using an automatic vernier caliper at 0.01 cm precision and recorded. The shell mass was recorded on a balance to the nearest 0.1 g. The recorded biometrics were then used to select 44 live- and 30 dead-collected shells for sectioning. The selected shells were those which were the largest by height, and the heaviest by total shell mass, chosen on the basis that these were likely to be the longest-lived (see [10]). Shells which were damaged by the dredge or fouled were excluded.

The selected live- and dead-collected shells were sectioned using the methods described by Ramsay et al [43]. A 3–4 cm section was cut from the hinge through to the ventral margin along the axis of maximum growth using a diamond tipped blade mounted on a rotary grinding saw, making sure that the apex of the umbone was included in the section. The cut section was then embedded into Escil polyester resin and placed in an oven set at 30°C until dry before a final section was cut with a precision saw (Struers Secotom-10) along the axis of maximum growth. The cut surface was ground using silicon carbon paper (grades 800–4000) fixed to a mechanical grinding machine (Struers TegraPol-35) before being polished using 3μm diamond paste. The polished shell sections were then etched in 0.1M HCl for 90 seconds, soaked in a water bath and left to air dry [16, 35, 43]. Acetate peel replicas of the etched surfaces were then produced using methods described by Richardson [20].

**Fig 2:**
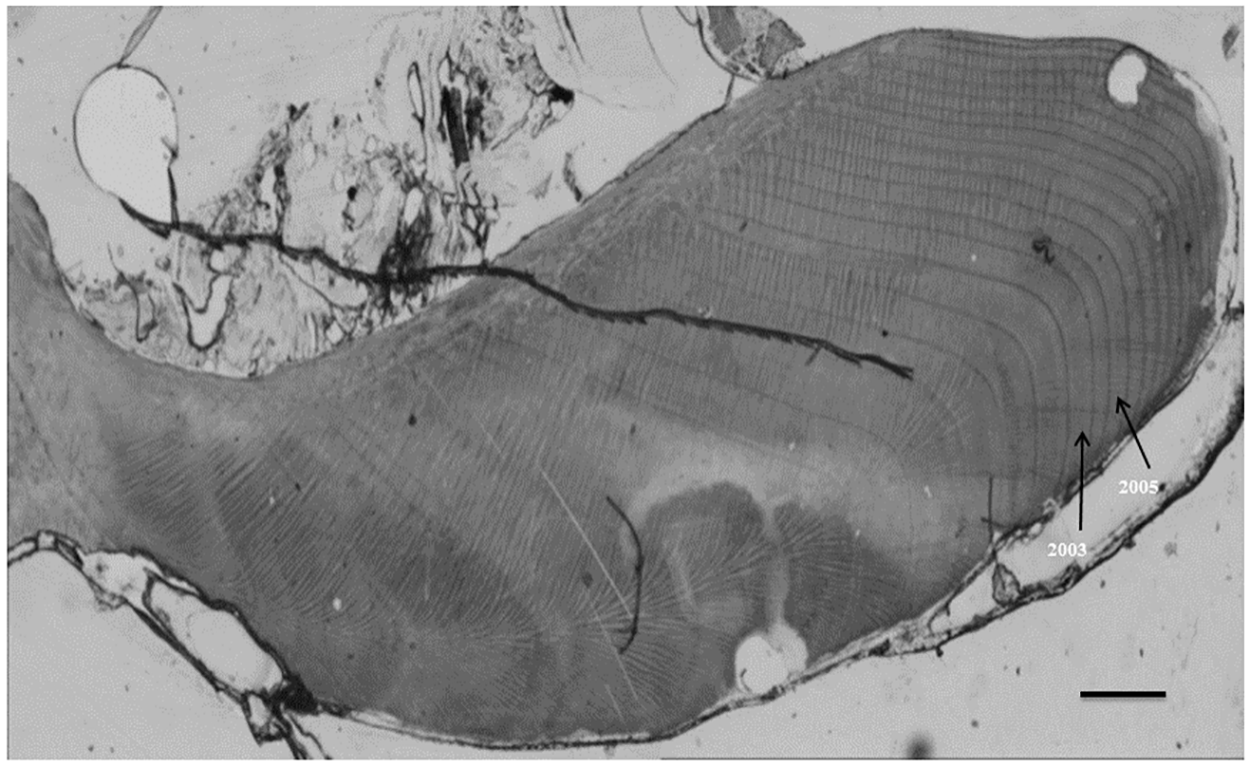
A digital photomosaic of an acetate peel replica of growth increments in the *G*. *glycymeris* hinge. Black scale bar is 500μm. Black arrows indicate marker years where there was increased growth. Shell ID: 14101004, collected in October 2014.

The prepared acetate peels were digitally photographed with a Zeiss AxioCam MRc5 digital camera mounted on a Zeiss Lumar.V12 light-transmitting microscope under 40 and 80× magnification (Fig 2). The software Axiovision V4.9.1 was used to create photo mosaics from the individual photographs, and the growth increments seen in the images were crossdated visually using the list year method [44]. This technique is based on the assumption of synchronous growth in individuals sampled from the same area [44, 45]. The growth increments were then digitally measured, using ImageJ software. Because the increment widths in *G*. *glycymeris* typically show little variability, a mean value was taken of three sets of measurements in order to minimize the effect of measurement error [33]. The growth measurements were taken in the hinge rather than the ventral margin as the hinge provides a more consistent orthogonal transect through the increments [35].

### Chronology construction

Standard statistical techniques derived from dendrochronology were used to crossdate the growth increment series [12, 13, 16, 17, 33, 46]. Only those samples within which the growth increments were well defined and which were taken from individuals that were over 20 years old were used for chronology development. In total 20 live-collected and 18 dead-collected individuals satisfied these criteria. The year of collection (2012, 2014 or 2015) was assigned to the most recent partial growth increment of the live-collected specimens; this is the incomplete increment found on the outer edge of the hinge.

The dendrochronological crossdating application COFECHA V6.06P [47, 48] was used to crossdate the growth increments between different individuals. For the dead collected specimens, where the date of death was unknown, 2004 was initially assigned to the most recent growth increment so that the series could be input to COFECHA. COFECHA was configured to fit a 20-year cubic smoothing spline with 50% wavelength cutoff to the measured time series. This 20-year smoothing was chosen due to the young average age of the specimens included in the analysis along with the standard 50% wavelength cut-off. Each time series was then divided by the values predicted by the spline, isolating high-frequency variability and standardizing each series to a mean of one [48]. The overall average of the correlations between each individual and the average of all others was reported as the series intercorrelation. The dead collected individuals were reassigned new arbitrary ages at ten-year intervals until COFECHA could give a potential fit. Once a fit was found the list year method was used to check that the match was also visually correct. The successfully crossdated shell series were used to construct a master chronology using the dendrochronology application ARSTAN for Windows (version 41d, [49]).

We used detrending methods that have been previously applied in sclerochronological studies to produce *G*. *glycymeris* [16] and *G*. *pilosa* chronologies [17]. We first applied an adaptive power transformation to each series to stabilise variance throughout the growth series [50]. Once this was completed, a negative exponential function was fit to the transformed series to remove the ontogenetic growth curve. In 6 cases, however, ARSTAN indicated that a 15-year cubic spline with 50% frequency cut off provided the best fit [51] by announcing that the previous function would lead to a negative detrended growth curve. ARSTAN creates three versions of the master chronology (standardized (STD), residual (RES) and ARSTAN (ARS)), which model autoregression in different ways. In this study, there were no significant differences between the three versions, so only the STD chronology will be used in the remainder of this analysis.

### Chronology validation

Accelerator mass spectrometry (AMS) radiocarbon (^14^C) dating was used to validate the crossdating between four dead-collected *G*. *glycymeris* that cross matched with each other but could not be visually incorporated into the master chronology. CaCO_3_ samples from all four shells, drilled close to the ventral margin, were sent to Beta Analytic, Miami, USA, for analysis. Conventional Δ^14^C determinations were corrected for a regional marine radiocarbon reservoir age effect (MRRE) using0020ΔR of −48 ± 45 years [52] and, as all samples were post-bomb (post-1950), they were calibrated using regional bomb-pulse calibration curves created by Scourse et al [46], on the basis of the marine box model used by Reimer et al [53]. Shallow, well-mixed, locations like the Bay of Brest have a response similar to the German Bight or Oyster Ground which approximate the atmospheric bomb-pulse because carbon is readily exchanged and mixed in such settings. As such the pMC (% modern carbon) values were compared with the German Bight and Oyster Ground curves found in Scourse et al [46].

### Environmental analysis

The STD chronology standardized growth index (SGI) was compared to available variables from the local SOMLIT-Brest monitoring station, as well as the North Atlantic Oscillation (NAO). The NAO is an index of fluctuations in atmospheric pressure at sea level between the subpolar and subtropical regions [54]. It is an indicator of weather patterns (wind, temperature, moisture, etc.) in the North Atlantic, especially the strength and direction of westerly winds and storm tracks during the winter months [55]. The winter NAO index used here is defined as the normalized pressure difference between the Azores (high-pressure) and Iceland (low-pressure) [54], averaged over the months December-February.

The same winter months were averaged for the East Atlantic Pattern (EAP) and compared to the SGI. The EAP has a strong impact in Western Europe by influencing sea surface temperature or modulating mean precipitation rates and hydrological processes [56].

The SGI was compared with temperature, salinity and chlorophyll α data acquired by the SOMLIT-Brest monitoring station from 1998 to 2014. Further comparisons were made between the SGI and rainfall using a dataset from Brest-Guipavas first published by Klein Tank et al [57].

Data about the river flow rates of both the Elorn and the Aulne, nitrite and suspended particulate matter (SPM) was provided by Hydro Bank, which is administered by the Service Central d’Hydrométéorologie et d’Appui à la Prévision des Inondations (service du Ministère de l’Ecologie, du Développement Durable et de l’Energie). This data can be found at http://www.hydro.eaufrance.fr/.

The correlating environmental data were standardised (μ = 0, σ^2^ = 1) and analysed using principal components analysis (PCA). Missing values were corrected using a mean value imputation. The scores for the principal components that accounted for the majority of the variance (PC1, PC2 and PC3) were tested for significant correlations (using Pearson’s correlation) with the SGI.

## Results

### Biometrics and growth

In total 401 live- and 243 dead-collected *G*. *glycymeris* with paired valves were collected between September 2014 and November 2015. The mean shell length of all specimens collected was 59.2 mm (σ = 9.3 mm). The mean length of the dead-collected valves was 61.8mm (σ = 6.9 mm; range 34.6 mm to 77.1 mm), and that of the live-collected valves was 52.5 mm (σ = 8.2 mm; range between 24.6 mm and 69.4 mm). There was also a difference between the shell mass of the live- and dead-collected shells, the overall average being 42.4 g (σ = 19.4 g). The dead-collected paired valves weighed an average 55.3 g (σ = 14.7 g; range 8.6 g to 114.8 g), while the live-collected shells weighed much less with an average of only 34.62 g (σ = 14.1 g; range 3.1 g to 95.9 g).

The age ranges of the live-collected shells was 5 to 43 years, with an average of 19 years (σ = 9 years). This is low compared to the ages of the shells live-collected in 2012, whose average age was 27 years (σ = 9 years; range 25 years to 44 years). The dead collected shells had a greater longevity with an average of 44 years (σ = 17 years; range 24 years to 70 years). The maximum age of live-collected specimens from all of the 2015, 2014 and 2012 collections was 50 years, whereas the longest lived dead-collected valve analysed was 70 years. The mean longevity of the shells used to construct the chronology was 32 years.

### Chronology

*G*. *glycymeris* has strong synchronous growth that allows it to be crossdated. Signature years of increased growth, such as 2005, 2003, 2001, 1995 and 1983, were found in almost all samples. No missing increments were observed in any sample. Non-annual growth lines were present in all sampled individuals, but these were easily distinguished from the annual increments as they were always lighter in colour than the annual banding.

**Fig 3:**
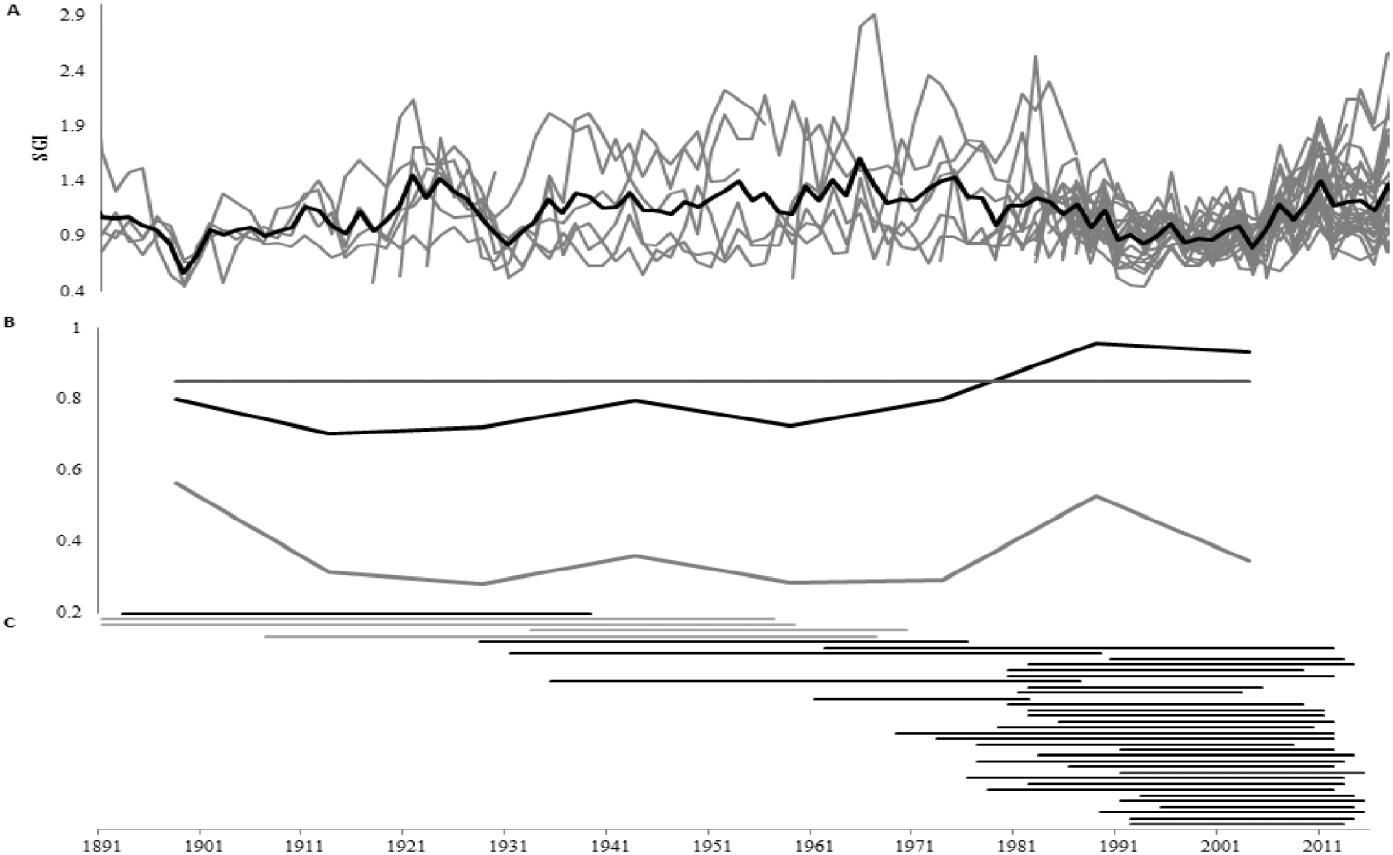
**A)** Detrended increment measurements (grey lines) and ARSTAN created standardized (STD) chronology (black line); **B)** EPS and RBar values calculated in a 20 year window with a seven year overlap. The horizontal line shows the 0.85 threshold (see text); **C)** The position of each of the shells in the chronology. The grey lines indicate shells sent for radiocarbon analysis.

The chronology created with only live-collected specimens has a mean sensitivity of 0.189, with an interseries correlation of 0.497. When the dead collected shells are added to extend the chronology further back in time, the mean sensitivity remains almost identical at 0.190, and the interseries correlation rises slightly to 0.502. Using only live-collected individuals, the replicated chronology extends from 1975 to 2015, with five individuals in the chronology at 1975 (Fig 3C). When series from the dead collected shells are added, the replicated chronology extends back to 1901 with at least 3 individuals. One specimen reaches back to 1891. The Expressed Population Signal (EPS), a measure of chronology strength, falls below the conventional threshold of 0.85 [58]; (see Discussion for further explanation) prior to 1975 because of the rapid fall in sample depth as live-collected specimens drop out of the chronology, but remains above 0.7 throughout. The chronology was truncated at 1901 because the running EPS (calculated over a 20 year window with a 7 year overlap) could not be calculated before that point (Fig 3C).

### Chronology validation

The four AMS ^14^C determinations derived from the ventral margins of shells were dated as post-bomb (post 1950) and were therefore calibrated using the curves for German Bight and Oyster Ground described by Scourse et al [46]. The calibration indicated that the individuals had died c.1960 or post 2005 (S1 Table), confirming their placement in the master chronology between 1955 and 1970.

### Environmental drivers

**Fig 4:**
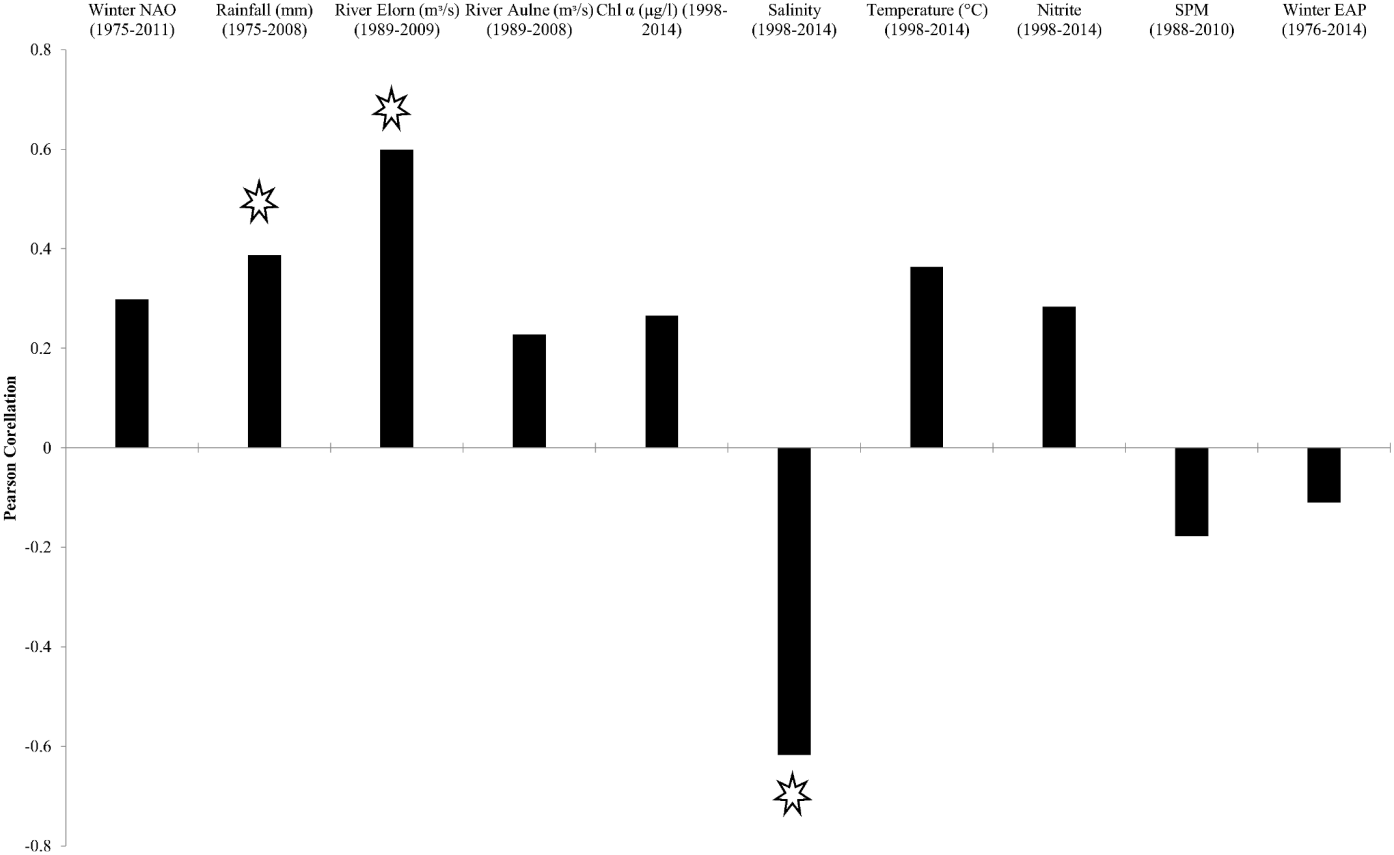
Pearson correlation coefficients of annually averaged environmental data with SGI. Significant correlations (p < 0.05) are marked with a star.

In the Bay of Brest, growth of *G*. *glycymeris* was negatively correlated to annually averaged salinity (r = -0.62, p = 0.006) over the period 1998–2014. Growth was positively correlated with the annually averaged flow rate of the River Elorn (r = 0.60, p = 0.005) over the period, 1989–2009 and with rainfall (r = 0.34, p = 0.03) over the period 1975–2008 (Fig 4). No significant correlations were found with any other annually averaged variable.

**Fig 5:**
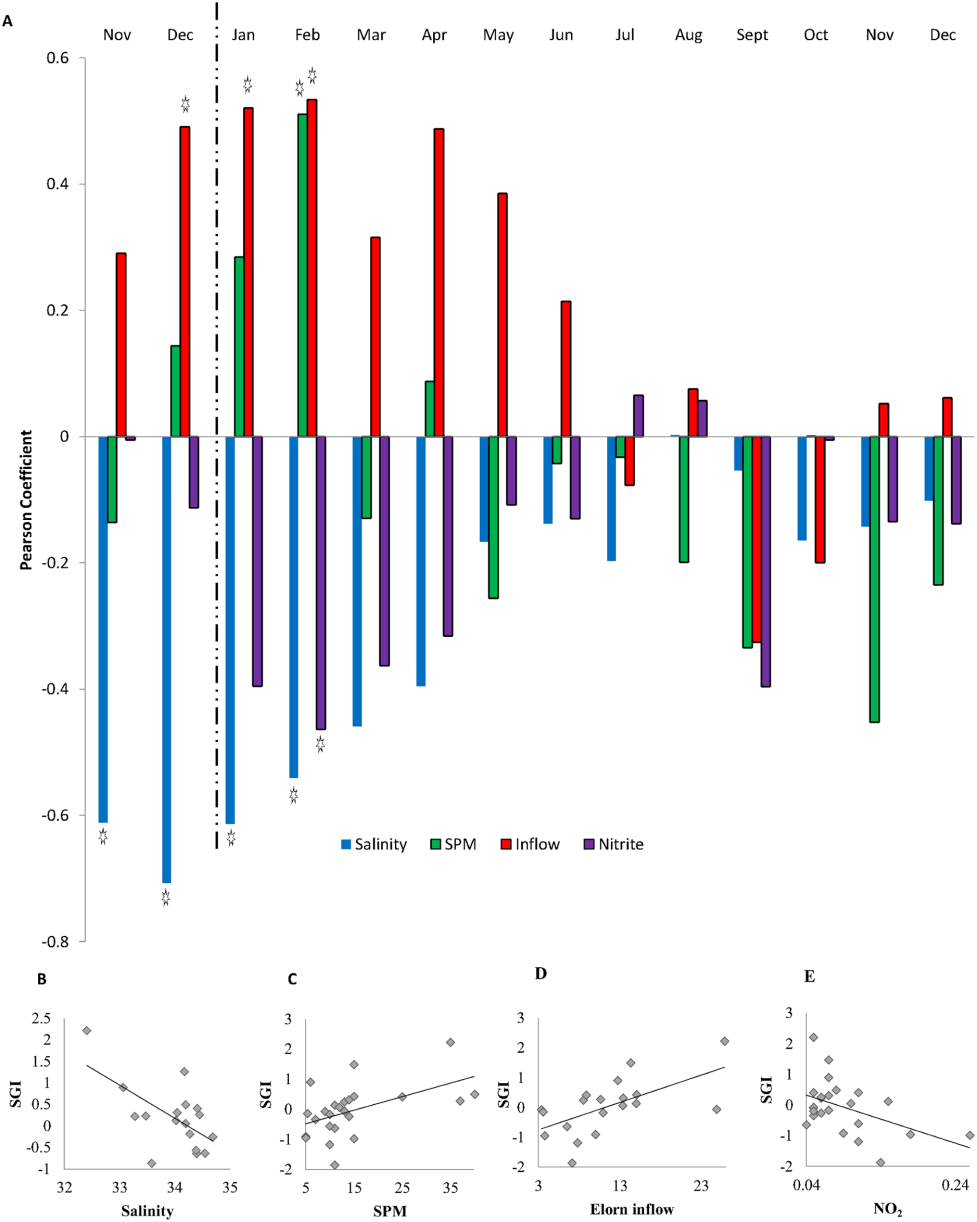
Comparison of chronology with environmental data. **A)** Pearson correlation coefficients of monthly environmental data with the SGI. Significant correlations (p < 0.05) are marked with a star. Dotted line indicates the change between years (e.g. previous and current year). **B-E)** biplots of significant environmental data (averaged monthly) and the chronology.

When the monthly averages of each environmental variable were correlated with the chronology, the strongest correlations with salinity were for the months of January (r = -0.62, p = 0.008) and February (r = -0.54, p = 0.020), and also for November (r = -0.61, p = 0.01) and December (r = -0.71, p = 0.001) of the previous year. The months for which SGIs correlated most strongly with inflow of the River Elorn were January (r = 0.52, p = 0.022) and February (r = 0.53, p = 0.019) as well as December of the previous year (r = 0.49, p = 0.033) (Fig 5). In February alone, there were significant correlations with nitrite concentration (NO_2_) (r = -0.464, p = 0.022) and suspended particulate matter (SPM) (r = 0.500, p = 0.01) (Fig 5).

### Principal components analysis

**Fig 6:**
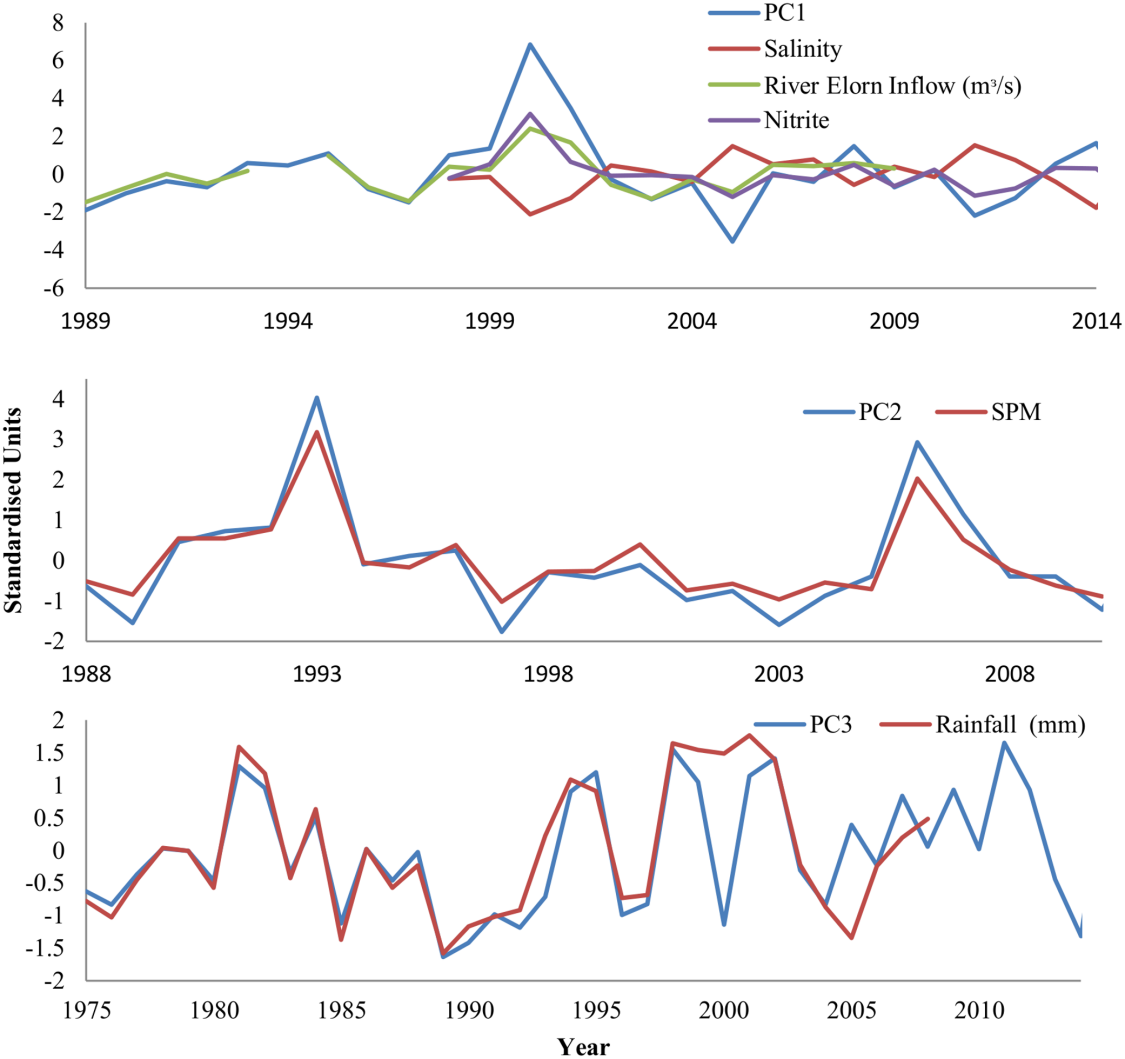
Environmental data with the respective leading principle component (PC) scores. Environmental parameters included in analysis are: salinity, River Elorn inflow rate, nitrite levels and rainfall. Each environmental parameter was normalised (mean = 0, variance = 1).

The standardised environmental parameters of nitrite, River Elorn inflow, salinity and rainfall were analysed using a PCA. The first principle component (PC1) accounted for 51.9% of the variance, PC2 accounted for 21.2% and PC3 of 17.7%. PC4 and PC5 accounted for less than 15% of the variance and were subsequently disregarded from further analysis (S2 Table). Two of the environmental parameters had similar strength loadings on PC1 (River Elorn Inflow; 0.51; nitrite, 0.55). Another strong loading in PC1 was salinity (-0.52) although it was a negative loading. SPM was the strongest loading on PC2 (0.93) and rainfall was strongest in PC3, showing strong synchrony between these environmental factors and their respective principle components (Fig 6 (see S3 Table for all loadings)). PC1 was positively correlated with the SGI (r = 0.35, p = 0.02) whereas neither PC2 (r = -0.12, p = 0.44) or PC3 (r = 0.17, p = 0.29) were found to have significant correlations.

## Discussion

In this study we examined the internal growth increment series in shells of the marine bivalve *G*. *glycymeris* collected from the Bay of Brest, France. Series from 20 live- and 18 dead-collected shells were successfully crossdated to construct a replicated chronology extending from 1901 to 2014. The longest-lived shell analyzed was a dead collected individual whose longevity was estimated at 70 years, making it the longest-lived individual of this species so far found in north-west France (Royer et al [26] have previously reported a maximum longevity of 46 years). It is interesting to note that other studies, conducted at higher latitudes, found significantly greater longevity in *G*. *glycymeris* [16, 33, 35], with Reynolds et al [16] identifying a specimen that had lived for 192 years. Reynolds et al [16] collected their samples from north-west Scotland, whereas Ramsay et al [35] and Brocas et al [33] sampled the waters surrounding the Isle of Man. This supports the hypothesis that there is a latitudinal trend, with longevity increasing at higher latitudes. Moss et al [59] found this effect in many different bivalves including species from the commercially important genera *Veneridae*, *Pectinidae*, and *Mytilidae* and attributed it to differences in temperature and the limited and highly seasonal food availability that affects populations at higher latitudes. They theorise that the longer lifespan may be a consequence of limited metabolism, and that long life could be the key to reproduction because with limited food availability individuals could not always rely on the energy resources to allow annual spawning.

Wigley et al [58] determined that an EPS of 0.85 indicates that the signal in the chronology is a sufficiently good representation of the signal in the whole population. This chronology achieves that threshold after 1975, but because of the reduced sample depth, EPS drops below 0.85 before that date, sometimes falling as low as 0.7. Since all the comparisons with environmental data here relate to the period after 1975, the chronology signal strength can be considered adequate for these analyses. The use of this chronology as a robust reconstruction tool is contingent on its being strengthened prior to 1975 with the addition of growth increment series from more shells.

The significant correlation observed between *G*. *glycymeris* growth and the inflow of the River Elorn is to be expected. The rivers feeding into the Bay of Brest are a substantial source of nutrients that sustain a large amount of primary productivity in the bay [56, 60]. Although the River Elorn is the smaller of the two rivers, it is closer to the sample site than the larger River Aulne, and it is therefore reasonable to conclude that it might have a more direct influence on the growth of this *G*. *glycymeris* population. Although a significant negative correlation was found between growth and salinity, it is unlikely that there is a direct relationship between the change in salinity and growth. Colonese et al [61] found a similar relationship between growth, freshwater circulation and salinity using intra-shell δ^18^O and δ^13^C values of the freshwater mollusc *Anomalocardia flexuosa* from southern Brazil. However, *G*. *glycymeris* is a marine stenohaline species with an optimum salinity at 34 [62], so it is unlikely that an increase in salinity would cause growth to decrease, especially as average salinity in the Bay of Brest is below the upper tolerance limit for the species and very close to the optimum. It can therefore be assumed that another variable, also related to river inflow, is controlling growth. Del Amo et al [63] reported correlations (Spearman’s rank correlation) between concentrations of silicic acid and phosphate and river inflows during 1993–94 that were higher for the Elorn than for the Aulne. The long term trend to lower Si:N molar ratios [39] has resulted in silicates and phosphates, rather than nitrates, being the main limiting nutrients in the Bay of Brest [60, 63]. It is therefore feasible that the Elorn is more significant than the Aulne in the delivery of limiting nutrients to the Bay of Brest, so that shell growth is more sensitive to changes in inflow from the Elorn. This hypothesis should, however, be treated with caution as it is based on measurements for a single annual cycle.

The strong positive correlation between growth and suspended particulate matter (SPM) in February supports the hypothesis that food availability is an important driver of shell growth in the Bay of Brest. As *G*. *glycymeris* is part of the endofauna, living below the surface of the sediment, the predominant effect of nutrients on shell growth must occur after the particles settle. Active pumping and biodeposition by benthic suspension feeders have been found to increase the rate of settlement of suspended matter on the sediment [64]. For example, De Vries & Hopstaken [65] have previously estimated, for Grevelingen (The Netherlands), that biodeposition by benthic suspension feeders increases particulate matter settlement by at least three times compared to passive sedimentation [66]. In the Bay of Brest, Barnes et al [67] found that biodeposition by *Crepidula fornicata* led to siltation of sediment as well as significantly reduced particle resuspension. This activity by *C*. *fornicata* appears to be a crucial factor in the development of a silicate pump in the Bay of Brest, with the biologically limiting silicates retained in the bay as a result of such biodeposition and contributing to diatom dominated phytoplankton blooms during the spring and summer [66, 68]. *C*. *fornicata* is found in great numbers (500–1300 individuals/m^-2^) close to the sample site of this study [55, 66], and it is likely that the retention of nutrients in the area through biodeposition is directly related to the availability of nutrients to *G*. *glycymeris* at this site.

High levels of chlorophyll α are not necessarily correlated with high growth rates in bivalves [69]. Lorrain et al [70] observed that large bottom concentrations of chlorophyll α, particularly after diatom blooms, could have a negative effect on the ingestion or respiration of *P*. *maximus* juveniles, either by gill clogging or by oxygen depletion at the water-sediment interface associated with the degradation of organic matter. *G*. *glycymeris* has a ciliated gill structure which allows for potential food to be sorted upon the gills themselves, rather than through the digestion process and unwanted material is passed to the edges of the gills demibranchs [71]. This structure of constant sorting and movement of particles along the gills means that gill clogging is more likely than in species with a lophophore or siphon [72].

Overall, phytoplankton is thought to be only a small part of the diet of *G*. *glycymeris* [34] and therefore SPM is likely a much better representation of food availability for this species within the normal seasonal cycle. This is not the first time such a conclusion has been made. Galap et al [73] states that bacteria enriched detritus, collected from the sediment, constitutes as the principle nutrient source for *G*. *glycymeris* in the Douarnenez Bay, France. Also, this is not restricted to *G*. *glycymeris* in France, the same has been observed in Mali Ston Bay, Croatia, where the main food source of *G*. *nummaria* is detritus, particularly in the autumn/winter months [74].

The negative correlation observed with nitrite (NO_2_) is likely due to the high toxicity of the compound. Widman et al [75] found that, after ionised ammonia, NO_2_ was the most toxic nitrogen based compound for *Argopecten irradians irradians*. Argumugan et al [76] observed that *Mytilus galloprovinciais* and *Crassostrea gigas* produce NO_2_ as a by-product of their reactive oxygen intermediates, although these are the only species found to do so. While *Mercenaria mercenaria* and *C*. *virginica* have been shown to have a strong tolerance for nitrite [77], the same has not been observed in *G*. *glycymeris*. More research needs to be carried out on the *Glycymeris* genera as up until now there have been no studies of their response to environmental NO_2_. This is particularly important in areas such as the Bay of Brest, where inflows of nitrogen compounds have increased ten times over the course of the 20^th^ century [78].

The addition of principle components analysis (PCA) related nitrite levels to the inflow from the River Elorn as well as finding a negative relationship to salinity (S3 Table) grouping them together for analysis within PC1. This leads to the conclusion that the nitrite levels in the Bay of Brest are being fed by the River Elorn. As this grouping correlated strongly with the SGI, it can be concluded that all of these factors are driven together and that they influence the growth of *G*. *glycymeris*. More information is needed to pull apart exactly which of these environmental factors is the most important. Although correlations were found with SPM levels in February (Fig 5), the lack of correlation between the SGI and PC2 implies that this correlation may be coincidental.

In contrast with other bivalve growth studies [33, 79], no significant correlation was found between the growth of *G*. *glycymeris* and the winter NAO (wNAO). The wNAO reflects winter conditions in Northern Europe, as it indicates the direction of storms caused by winds blowing across the Atlantic [80]. Royer et al [26] found that the growing season for *G*. *glycymeris* in the Bay of Brest is May-October, and is therefore lagging the period of maximum pressure gradients in the North Atlantic (upon which the wNAO index is based) by several months. The same authors also found no correlation between the wNAO and growth in their population from the Chausey Islands, and concluded that this was because of the time lag. However, it has been suggested by Schöne et al [79] that the state of the NAO in winter could predispose the environment to favour shell growth later. Correlations between the wNAO and shell growth, even if they are only intermittent [30], seem to occur at higher latitudes than the populations in NW French waters investigated by Royer et al [26] and in this study. This suggests that the effect of the positive phase of the wNAO is to divert the Atlantic storm tracks into UK and Norwegian waters. The result is that the lagged signal of the wNAO is more strongly expressed in bivalve populations living directly under the path of the stronger storms that occur when the wNAO is in its positive phase. For the *G*. *glycymeris* population studied here, it is likely that local factors are more important for growth than major climate oscillations. For example, Grall and Glémarec [81] describe the river Elorn as being heavily polluted by agricultural runoff and this may lead to larger scale environmental signals such as the NAO being masked in the shell growth records [82].

Tréguer et al [55] found a positive correlation between sea surface salinity, rainfall and the East Atlantic Pattern (EAP) in the Bay of Brest. It could be assumed that, because the growth rate of *G*. *glycymeris* in this location is controlled by rainfall and river runoff (indirect correlation with salinity), it would be linked with the EAP as well. However, our study shows that this is not the case. Tréguer et al [55] only used data spanning fifteen years, on the other hand this study utilised almost forty years by comparing the EAP with the created SGI, going back further in time than salinity observations within the bay allow. The difference between the results in this paper and those by Tréguer et al [55] lends weight to the requirement of longer proxies in order to establish significant climatic trends [83].

### Conclusions

This study indicates that *Glycymeris glycymeris* in the Bay of Brest is highly sensitive to the fresh water inflow from the River Elorn, as well as to food availability mediated by increased SPM in the late winter. As the length of the chronology extends to periods before measured environmental data was available, it will be possible to reconstruct such variables using the chronology SGIs, subject to improvement of the chronology signal (EPS) by adding more shells to the chronology before the 1970s. It will also be possible to integrate other biological data (such as fish otolith chronologies, changes in benthic species composition, and phytoplankton observations) into this research, using mixed effects models to test the relationships between local environmental variables and different combinations of ecological and biological responses. Given the large quantities of fossil material available in the relatively small area sampled for this study, we are confident that a robust chronology extending further back in time can be constructed. In addition, with the use of radiocarbon dating, we will be able to construct floating chronologies for earlier periods of climatic and environmental interest. This study highlights the importance of location as a factor in the degree to which individual growth in a population responds to climatic and environmental change. Localised records such as these have great potential for the calibration of regional climate models as they provide unique sources of annually-resolved and locality-specific palaeoclimate information that is often not available from instrumental measurements.

## Supporting information

S1 Table pMC, Fraction modern and Δ^14^C of four *G*. *glycymeris* shells. (PDF)

S2 Table Correlation matrix of annual averaged environmental data. (PDF)

S3 Table Loadings of the environmental data on principal components. (PDF)
