# Supplementary material for: Influence of riverine input on the growth of Glycymeris glycymeris in the Bay of Brest, North-West France
Source: PLoS One. 2017 Dec 20;12(12):e0189782. doi: 10.1371/journal.pone.0189782 (PMC5738111; doi:10.1371/journal.pone.0189782)
Supplement: S1 Table — (PDF) [file pone.0189782.s001.pdf]

**Percent Modern Carbon (pMC)**	**Fraction Modern**	**$\Delta^{14}C$**
105.2 +/- 0.3 pMC	1.0524 +/- 0.0026	52.4 +/- 2.6 o/oo
96.9 +/- 0.4 pMC	0.9694 +/- 0.0036	-30.6 +/- 3.6 o/oo
99.6 +/- 0.4 pMC	0.9963 +/- 0.0037	-3.7 +/- 3.7 o/oo
102.6 +/- 0.3 pMC	1.0265 +/- 0.0026	26.5 +/- 2.6 o/oo
