# Supplementary material for: Influence of riverine input on the growth of Glycymeris glycymeris in the Bay of Brest, North-West France
Source: PLoS One. 2017 Dec 20;12(12):e0189782. doi: 10.1371/journal.pone.0189782 (PMC5738111; doi:10.1371/journal.pone.0189782)
Supplement: S2 Table — (PDF) [file pone.0189782.s002.pdf]

PC	Eigenvalue	% variance
1	2.59279	51.856
2	1.05997	21.199
3	0.884272	17.685
4	0.310491	6.2098
5	0.15248	3.0496
