# Supplementary material for: Influence of riverine input on the growth of Glycymeris glycymeris in the Bay of Brest, North-West France
Source: PLoS One. 2017 Dec 20;12(12):e0189782. doi: 10.1371/journal.pone.0189782 (PMC5738111; doi:10.1371/journal.pone.0189782)
Supplement: S3 Table — (PDF) [file pone.0189782.s003.pdf]

	PC 1	PC 2	PC 3	PC 4	PC 5
**Rainfall**	-0.0011988	0.11132	0.12235	0.98621	-0.0058181
**River Elorn**	0.057075	0.67754	0.71444	-1.65E-01	0.0050399
**Nitrite**	0.019785	0.72249	-6.86E-01	4.10E-03	0.080518
**Salinity**	0.00027532	-0.061256	0.05249	6.28E-03	0.99672
**SPM**	0.99817	-0.052912	-0.027114	0.010537	-0.002166
